# Supplementary material for: WordCommentsAnalyzer: A windows software tool for qualitative research
Source: F1000Res. 2018 Sep 4;7:536. Originally published 2018 May 3. [Version 2] doi: 10.12688/f1000research.14819.2 (PMC6137411; doi:10.12688/f1000research.14819.2)
Supplement: Supplementary file 3 [file f1000research-7-17614-s0002.tgz › b2a79496-4ada-47c4-9682-a99ce867836d.zip!/Eight Iranian Health Journals/Journal of Research in Rehabilitation Sciences _18.docx]

© Journal of Research in Rehabilitation Sciences . This work is licensed under CC BY-NC 4.0 (https://creativecommons.org/licenses/by-nc/4.0/).

Volume & Issue: ويژه نامه ورزشي، دوره 10، شماره 7، 1393

Title: بررسی تاثیر شش هفته تمرین منتخب تعادلی بر سرعت تحرک و تعادل بیماران ام اس

http://jrrs.mui.ac.ir/index.php/jrrs/article/view/1280

چکیده مقدمه:   بیماری مالتیپل اسکلروسیس (MS)یکی از شایع ترین بیماری های سیستم اعصاب مرکزی است که در اثر تخریب غلاف میلین ایجاد می شود. ام اس باعث تخریب میلین اعصاب مرکزی می شود که می تواند موجب ایجاد اشکال در انتقال پتانسیل عمل در زمانی شود که سیستم عصبی مرکزی قصد دارد با بدن ارتباط برقرار کند و بر عکس.  هدف این تحقیق تاثیر شش هفته تمرین منتخب تعادلی بر سرعت راه رفتن و تعادل بیماران مبتلا به ام اس بود.  مواد و روشها: در این تحقیق  30 بیمار مبتلاء به ام اس با ناتوانی کم تا متوسط ، بر اساس مقیاس ناتوانی کورتزکی، انتخاب  و پس از انجام آزمون تعادلی برگ به دو گروه تجربی (15نفر) و کنترل (15نفر) تقسیم  شدند .سپس از هر دو گروه آزمون سرعت راه رفتن به عمل آمد. گروه تجربی سه روز در هفته به انجام تمرینات تعادلی منتخب می پرداخت و گروه کنترل به فعالیت های روزانه خود  ادامه می داد. در پایان از هر دو گروه آزمون سرعت راه رفتن و تعادل به عمل آمد. از آزمون  tمستقل جهت تجزیه وتحلیل داده ها استفاده شد .سطح معناداری 05/0 در نظر گرفته شد . يافته ها: نتیجه این تحقیق نشان داد که تمرینات تعادلی در بهبود تعادل و سرعت راه رفتن بیماران ام اس تأثیر دارد . بين دو گروه تجربي و کنترل تفاوت معني داري در آزمون تعادل (23/3t=،05/0P<)و آزمون سرعت راه رفتن (44/4-t=  ،  05/0P<)  مشاهده شد.   نتيجه گيري :نتایج این تحقیق نشان داد که تمرینات تعادلی در بهبود تعادل و سرعت راه رفتن بیماران ام اس موثر بوده است.

Title: اثر تصویرسازی¬ذهنی و تمرین¬بدنی بر تعادل ایستای بیماران مالتیپل اسکلروزیس

http://jrrs.mui.ac.ir/index.php/jrrs/article/view/1858

مقدمه: تصویرسازی­ذهنی از عوامل روانی تأثیرگذار در بهبود مهارت­های حرکتی می­باشد.هدف از این مطالعه بررسی تأثیر تصویرسازی ­ذهنی و تمرین­ بدنی بر تعادل ایستای بیماران MS بود.      مواد و روش ها: مطالعه حاضر نیمه تجربی و جامعه آماری شامل همه بیماران مرد MS شهر اهواز در سال 1391 بود(300 نفر).برای نمونه گیری پرسشنامه تصویرسازی بینایی و حسی-حرکتی (KVIQ) در بین جامعه مورد نظر از بین جامعه مورد نظر33 نفر با توانایی تصویرسازی یکسان و دارای اختلال متوسط در مقیاس گسترش یافته ناتوانی بیماری (EDSS) انتخاب شدند و به صورت تصادفی در سه گروه تصویرسازی­ذهنی-تمرین­بدنی(ترکیبی)(11نفر)، تصویرسازی­ذهنی(11نفر) و تمرین­بدنی(11نفر به عنوان گروه کنترل) قرار گرفتند.آزمون رومبرگ تعدیل یافته(MRBT) برای ارزیابی تعادل ایستا در پیش­آزمون، پس­آزمون و آزمون یادداری استفاده شد. برنامه تمرینی، 3 جلسه در هفته به مدت 6 هفته صورت گرفت. آزمون یادداری 2 هفته پس از آخرین جلسة تمرینی گرفته شد.      نتایج: بعد از بررسی نرمال بودن داده­ها و برابری واریانس­ها، داده­ها به روش آماری تحلیل واریانس یکراهه با اندازه­گیری تکراری، تحلیل واریانس یکراهه و آزمون تعقیبی توکی تحلیل شد. نتایج این مطالعه نشان داد که بین گروه­های مختلف تصویرسازی بعد از 18 جلسه مداخله در مرحله پس آزمون (01/0=P-value) و یادداری (001/0=P-value) تفاوت معنی­داری وجود دارد و در هر دو مرحله گروه ترکیبی در مقایسه با گروه­های تصویرسازی ذهنی و گروه کنترل اجرای بهتری داشتند.     بحث: باتوجه به نتایج تحقیق، تصویرسازی­ذهنی را می­توان به عنوان یک روش کاربردی برای عملکرد بهتر تعادل ایستای افراد مالتیپل اسکلروزیس محسوب نمود.

Title: الگوی راه رفتن در بیماران مبتلا به مولتیپل اسکلروزیس و اثر توانبخشی بر آن

http://jrrs.mui.ac.ir/index.php/jrrs/article/view/1809

مقدمه: اختلال در الگوي راه رفتن بيماران مبتلا به مولتیپل اسکلروزیس (MS) بسیار شايع است. با وجود تحقیقات زیاد در این زمینه، نتایج متناقض است و هنوز پروتکل درمانی موثر بر بهبود الگوی راه رفتن این بیماران ارائه نشده است. هدف از اجرای این تحقیق ارائه ویژگی ها و الگوهای مختلف راه رفتن در بیماران مبتلا به MS و بررسی پروتکل های درمانی و ارائه یک پروتکل درمانی مناسب برای بهبود الگوی راه رفتن این بیماران بود. روش اجرا: یک جستجوی الکترونیکی در وب سایت هایPubMed, Embase, and ISI Web of Knowledge انجام و تحقیقات منتشر شده بر ویژگی های الگوی راه رفتن و انواع پروتکل های درمانی مؤثر بر آناز سال های 1990 ـ 2014 استخراج گردید.الگوی راه رفتن بر اساس پارامترهای فضایی ـ مکانی، مقادیر کلینیکی از قبیل سرعت، زمان و مسافت پیموده شده و انواع پروتکل های درمانی مؤثر بر آن مطابق با نتایج پیشینارائه شد. یافته ها:ویژگی های الگوی راه رفتن بیماران در مقایسه با افراد سالم شامل کاهش در متغیرهای طول گام، طول استراید، کادنس، سرعت، حرکات مفصل، افزايش زمان دبل ساپورت، نامتقارني دردوره هاي زمانی ايستادن بر روي پاي راست و چپ و تغییرپذیری زیاد در اکثر پارامترهای الگوی راه رفتن بود. دامنه حرکتی مفاصل نیز در فازهای مختلف راه رفتن با کاهش و افزایش همراه بود.یک برنامه ی توانبخشی خاص، بر اساس آموزش قدرت می تواند منجر به بهبود عملکرد بیماران در راه رفتن، تعادل، ثبات مفصل زانو و کاهش ناهنجاریهای زانو شود. نتیجه گیری:انواع الگوهای راه رفتن اسپاستیک و آتاکسی مخچه ایی در این بیماران به وضوح مشاهده می شود. طراحی يك برنامه توانبخشي با توجه به افزایش قدرت عضلات اندام تحتانی بویژه عضله همسترینگ و تصحیح الگوی راه رفتن برای این بیماران توصیه می شود.

Title: مروری بر تاثیر اسب سواری درمانی در کودکان با اختلال اوتیسم

http://jrrs.mui.ac.ir/index.php/jrrs/article/view/2027

اختلال اوتیسم طبقه ای از اختلالات عصبی رشدی است که بر جنبه های بیشماری از عملکردهای روزانه ی کودک تاثیر گذار است و با علایمی چون مشکل در برقرای ارتباط، تعامل اجتماعی، عملکردهای شناختی، عملکردهای حسی حرکتی همراه می‌باشد. مداخلات متعددی در این حوزه صورت می‌گیرد که یکی از این مداخلات استفاده از حیوانات در درمان، شامل اسب سواری درمانی است. با توجه به سهولت برقراری ارتباط کودکان اوتیسم با حیوانات نسبت به انسانها، برآن شدیم که به بررسی تحقیقاتی که تا کنون در زمینه ی استفاده از این درمان در کودکان با اختلال اوتیسم انجام شده است بپردازیم.  به نظر می‌رسد اسب سواری درمانی تاثیرات مثبتی برعملکرد کودکان اوتیسم دارد که البته نبود یک پروتکل واحد برای اجرای این مداخلات نیاز به انجام پژوهشهای بیشتری در این زمینه برای ایجاد یک پروتکل درمانی نشانمی‌دهد.
